# Histological Analysis of Multiple Unilateral Testicular Tumors in Dogs

**DOI:** 10.3390/life15111772

**Published:** 2025-11-19

**Authors:** Mirosław Kuberka, Przemysław Prządka, Stanisław Dzimira

**Affiliations:** 1 Private Veterinary Clinic Kuberwet, Kazimierz Wlk Str. 3, 63-300 Pleszew, Poland; kubervet@gmail.com; 2 Department and Clinic of Surgery, Faculty of Veterinary Medicine, Wroclaw University of Environmental and Life Sciences, Grunwaldzki Square 51, 50-366 Wroclaw, Poland; przemyslaw.przadka@upwr.edu.pl; 3 Department of Pathology, Faculty of Veterinary Medicine, Wroclaw University of Environmental and Life Sciences, Norwid Str. 31, 50-375 Wroclaw, Poland

**Keywords:** dog, unilateral, multiple testicular tumors, seminoma, Sertoli cell tumor, interstitial cell tumor

## Abstract

Testicular tumors are, after skin tumors, the most common neoplasms in male dogs. Among all animals, these tumors occur most frequently within dogs. The etiology remains unclear, although the ectopic (non-scrotal) positioning of the testicles has an influence on tumor development. The most common types of testicular tumors include seminomas, Sertoli cell tumors, and interstitial (Leydig) cell tumors. The aim of this study was a retrospective evaluation of preserved material. A total of 326 cases of testicular tumors in dogs, diagnosed between 2017 and 2024, were analyzed. A histological analysis of multiple unilateral testicular tumors was conducted, and the frequency of occurrence was determined. 27 instances (8.28%) of multiple tumors within the same testicle were identified. The most recurrent combination was seminoma and interstitial cell tumors—12 cases (44.44%), followed by Sertoli cell and Leydig cell tumors—6 cases (22.22%), and seminoma and Sertoli cell tumors—6 cases (22.22%). In three cases, the presence of three tumors within a single testicle was observed (11.11%). In one case, double tumors were found within both testicles. It was observed that malignant features, as in cases of single testicular tumors, are rare.

## 1. Introduction

Testicular tumors rank second, after skin tumors, in terms of occurrence in male dogs [1]. Amongst all domesticated animals, these tumors occur in dogs most frequently [1,2,3]. Their occurrence is estimated at 5–15%, and according to some authors, even up to 27% [2,3]. The relatively high prevalence in dogs is influenced by their longer lifespan compared to other species and the significantly less common practice of preventive castration. Testicular tumors most often occur within elderly dogs over 10 years of age (typically between 7 and 12 years), usually unilaterally, although bilateral localization is not uncommon [3,4,5]. The presence of histologically different types of tumors within the same testicle has been observed both in humans [6,7,8,9,10,11] and in animals—dogs and stallions [3,4,5,12,13,14]. As with almost every type of spontaneous tumor, the etiology is not clearly defined, but ectopic testicular positioning undoubtedly has a significant impact on tumor development. It is estimated that the risk of neoplasia in undescended testicles increases 14-fold [15,16,17,18]. In humans, it has been proven that an increased risk of testicular tumor development is reported in syndromes associated with gonadal dysgenesis, such as androgen insensitivity syndrome or Klinefelter syndrome [19]. In dogs, various cases of tumor formation in testicles affected by disorders in sexual development have also been described [20,21,22]. The individual tumor types that may occur in the testicles originate from different histological structures. Hence, they differ in histological appearance. They vary in growth rate, malignancy, metastatic potential, and the possible accompanying clinical symptoms [7,23]. The latter results not only from the size and location of the tumor, but also from its potential hormonal activity. Germ cell tumors of the testis develop from primitive cells, which may differentiate along gonadal lines (seminomas–seminomata) or transform into populations of pluripotent cells (non-seminomas–nonseminomata). Pluripotent cells may remain within an undifferentiated state (embryonal carcinoma–carcinoma embryonale), differentiate towards extraembryonic tissues (yolk sac tumor, or choriocarcinoma), or somatic tissues (teratoma). The most commonly occurring primary testicular tumors in dogs derive from the germinal epithelium of the seminiferous tubules (seminomas–*seminomata*, SEM), Sertoli supporting cells (*sertolioma*, Sertoli Cell Tumor–SCT) and Leydig interstitial cells (*leydigoma,* Interstitial Cell Tumor–ICT). Particularly in old dogs, more than one type of tumor may sometimes occur in the same testis (collision tumors). All tumors can lead to pressure atrophy of the germinal epithelium [24,25,26]. These tumors are also diagnosed in men, although they are less common than in dogs; however, their incidence shows an upward trend. Testicular tumors account for 1 to 1.5% of all male neoplasms and 5% of urological tumors, with an incidence in Western populations of 3–10 new cases per 100,000 men per year [9,27,28]. They are the most common malignant tumors in young men, with peak incidence between the ages of 25 and 35 [29]. Cryptorchidism is also a predisposing factor in humans, but other causes of testicular tumors remain poorly understood in both species [25]. The model of studying testicular tumors within dogs, as well as comparing neoplastic processes, can also be promising for human medicine. This is due to humans and dogs sharing similarities in the histological build of tumors, as well as living environments [30].

The aim of the study was to determine the frequency of multiple unilateral testicular tumors in dogs, analyze their histological malignancy, and compare them with data on solitary lesions of the same types.

## 2. Materials and Methods

The testicles examined were obtained during routine surgical castration procedures, performed for medical reasons. Indications for surgery included: preventive castration, unilateral or bilateral cryptorchidism, enlargement of one or both testes, as well as palpable and/or ultrasound-detected deformities and proliferative changes. Samples were collected between 2017 and 2024. The testicles examined (one or, less frequently, two) came from 326 dogs of various ages and breeds, including mixed breeds. From the collected material, cases involving multiple (at least two) different types of tumors in one or both testicles were selected—a total of 27 cases. Cases involving multiple tumors of the same type or solitary neoplastic lesions accompanied by other pathological processes in the testicle or epididymis, such as hydrocele, orchitis, and/or epididymitis of known or unknown etiology, were excluded. Specimens were fixed within buffered 10% formalin, then embedded in paraffin. Material was cut into 3 μm sections, and using the routine hematoxylin-eosin (H&E) method, were stained. Samples were encased in Superfrost Plus slides (Epredia, China) then using xylene were deparaffinized, before being rehydrated within distilled water. Using Mayer’s hematoxylin, slides were incubated for 3 min, before being washed with 3 changes of tap water, or until blue staining ceased. Following this, the slides were counterstained for 10 s without rinsing, then two repetitions of 95% ethanol was used to dehydrate the slides. Then the slides underwent two repetitions of 100% ethanol and two acetone repetitions, Xylene was used to clear slides with two repetitions of 10 s for all specimens. Finally, the slides were mounted with cover slides. The same pathologist performed evaluations of all subjects. Microscopic analysis was completed using an Olympus BX53 (Olympus, Tokyo, Japan) light microscope alongside an Olympus UC90 camera (Olympus, Tokyo, Japan), utilizing cellSens Standard V.1 software (Olympus, Tokyo, Japan). Statistical analyses were performed using Statistica 13.3 (TIBCO Software Inc., Palo Alto, CA, USA). The appropriate statistical tests were selected based on the type and distribution of data. Data normality was assessed with the Shapiro–Wilk test. Differences between the study group and the population of dogs diagnosed with testicular tumours were evaluated using the chi-square test. Differences among various categories were analyzed using either the Mann–Whitney U test or the Kruskal–Wallis test followed by Dunn’s post hoc test. A significance level of *p* ≤ 0.05 was considered statistically significant.

## 3. Results

In the examined material of 326 dogs, consisting of one or, less frequently, both testes with pathological changes, sent for histopathological examination in the years 2007–2024, 27 cases (8.28%) showed the presence of two or more histologically different tumors within the same testicular tissue. The most recurrent combination was seminoma and interstitial cell tumors—12 cases (44.44%), followed by Sertoli cell and Leydig cell tumors—6 cases (22.22%), and seminoma and Sertoli cell tumors—6 cases (22.22%). In three cases, the presence of three tumors within a single testicle was observed (11.11%). In one case, double tumors were found within both testicles.

Cases of multiple tumors were found in dogs of various breeds, with the largest group being mixed breeds—8 individuals. The dogs ranged in age from 7 to 15 years (mean age: 10 years), with the vast majority being 10 years or older—21 individuals. In most cases, the affected testes were positioned normally within the scrotum (24 cases), with only three being undescended (abdominal or inguinal cryptorchidism). In the remaining 299 dogs, the following testicular tumors were identified: 115 (38.46%) seminomas, 111 (37.12%) interstitial (Leydig) cell tumors, and 55 (18.39%) Sertoli cell tumors. In addition to neoplastic lesions, 16 (5.35%) cases of orchitis and/or epididymitis and 2 (0.66%) cases of hydrocele were also diagnosed. Among the 326 testes examined, 28 (8.58%) originated from abdominal or inguinal cryptorchid dogs. In the undescended testes, neoplastic lesions—predominantly Sertoli cell tumors—were observed in 21 (75%) cases, while the remaining specimens exhibited marked features of testicular atrophy. In 75 (23%) dogs, both testes were subjected to histopathological examination. Bilateral testicular neoplasms were detected in 26 (7.97%) of these patients.

**Figure 1:**
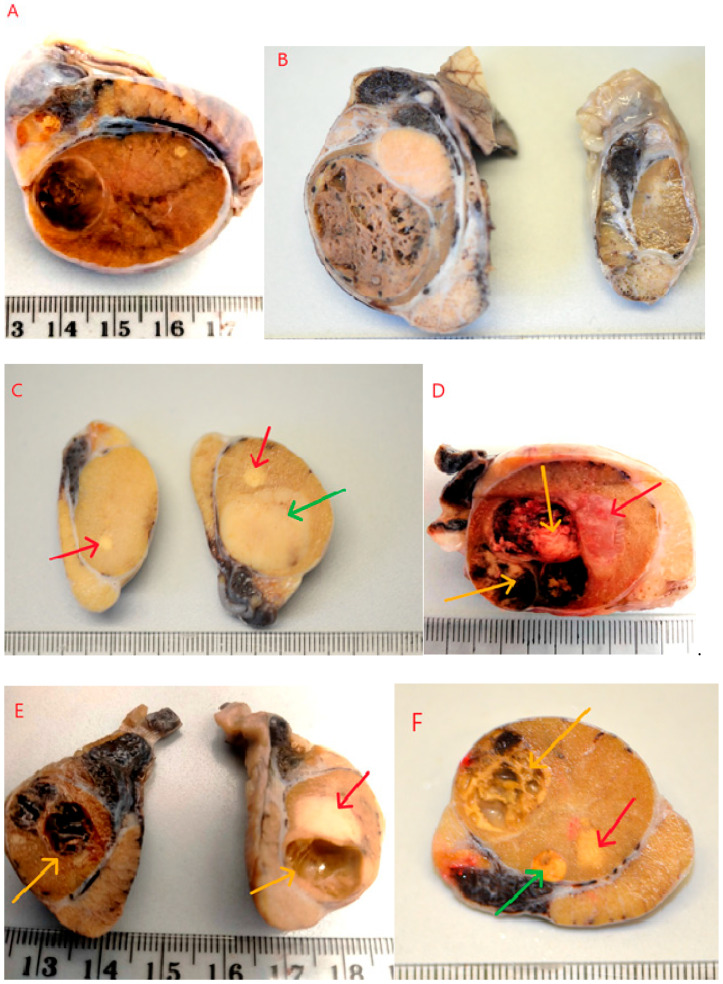
Macroscopic appearance of testes with multiple, diverse neoplastic lesions. (**A**)—Neoplastic changes that have not altered the contour of the testis. (**B**)—Enlarged testis with tumors on the left and an unaltered testis on the right. (**C**)—Multiple solid lesions in the right testis and a single lesion in the left testis. (**D**)—Hemorrhage and necrotic focus within a Leydig cell tumor. (**E**)—Solid and cystic lesions in both testes. (**F**)—Triple, distinct tumors within a single testis. Red arrows—SEM (seminoma), Green arrows—SCT (Sertoli cell tumor), Yellow arrows—ICT (interstitial/Leydig cell tumor). Scale in cm.

Testicles were cut along their longitudinal axis, revealing an altered internal structure. In most cases, macroscopically distinct or similar solid or cystic lesions were observed, sharply demarcated from the normal testicular parenchyma. In some instances, the lesions did not change the external contour of the testicle. As shown in Figure 1, some tumors did not cause deformation or enlargement of the testicle (Figure 1A). Such changes were often detected incidentally during sectioning of the testis, following preventive castration or identified preoperatively via ultrasound examination. In some cases, testicles affected by tumors were visibly enlarged compared to the contralateral testicle (Figure 1B). In the majority of cases, only one macroscopically altered testicle was submitted for histopathological examination. In 10 cases, both testicles were examined. In one of these cases, the multiple tumor process was bilateral (Figure 1E), and in three cases, multiple tumors were found in one testicle and a single tumor in the other (Figure 1C). In some instances, due to the presence of cysts or large hemorrhages (Figure 1D,E), the testicles were of a softer consistency. On palpation, a semi-fluid content and soft, less firm consistency of the testicle could be detected. On sectioning, solid lesions–usually two (23 cases) or three (4 cases) of varying size and color–were observed (Figure 1F). In addition to solid tumors, cystic areas filled with fluid or blood were visible (Figure 1D–F). Microscopic evaluation revealed combinations of the most commonly occurring tumor types: seminoma, Sertoli cell tumor, and/or Leydig cell tumor in various configuraions (Table 1).

**Figure 2:**
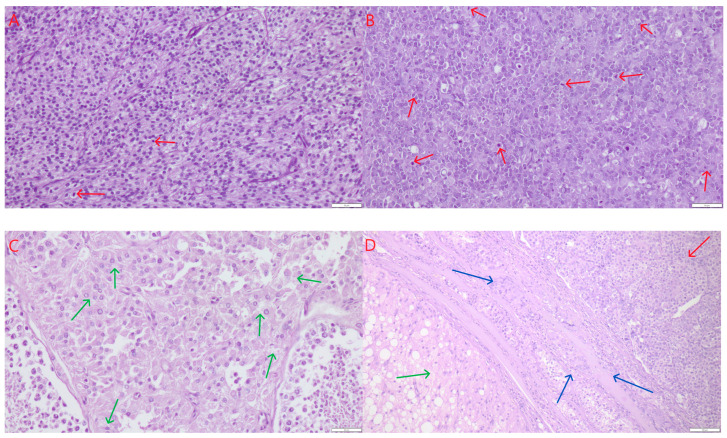
Microscopic appearance of testes with multiple, diverse neoplastic lesions. (**A**)—Intratubular type Sertoli cell tumor, with few mitotic figures (red arrows). (**B**)—Diffuse-type seminoma, with numerous mitotic figures (red arrows). (**C**)—leydigoma–round cells with vacuolated cytoplasm (green arrows). (**D**)—Interface (blue arrows) between seminoma (red arrow) and leydigoma (green arrow). HE stain, magnifications (**A**–**C**) 400×, (**D**) 200×. Scale bars standardized across images.

Sertoli cell tumors were found in combination with seminomas and Leydig cell tumors. Macroscopically, these appeared as nodules of varying sizes, ranging from 0.5 to 3.5 cm in diameter, occupying more than half of the testicle. They were white to whitish-gray in color, with a solid consistency, sometimes lobulated in appearance (Figure 1C,F, green arrows), and with clear demarcation from the testicular parenchyma. The neoplastic cells were elongated, with oval to spindle-shaped nuclei, arranged in palisade patterns, lining well-defined tubular structures. The connective tissue stroma was relatively abundant. Mitotic figures were rare, ranging from two to ten per 2.37 mm^2^ corresponds to 10 HPF-400× fields and an ocular FN 22 mm. The cytoplasm of the cells was finely granular, sometimes slightly vacuolated (small lipid vacuoles). Anisocytosis and anisokaryosis were minimally present (Figure 2A). No signs of fibrosis or necrosis were observed. In a few isolated cases, small hemorrhages were present.

Seminomas appeared macroscopically as solid, whitish, gray, or pale yellow tumors with diameters ranging from 0.5 to 3 cm. They occurred as single or multiple lesions, poorly demarcated from the surrounding testicular parenchyma (Figure 1C–F, red arrows). Occasionally, especially when larger, they exhibited microscopically visible foci of necrosis. Histologically, three growth patterns were observed: diffuse, intratubular-diffuse, and intratubular. The smallest lesions (up to 1 mm) typically exhibited the intratubular pattern. This in situ seminoma was characterized by atypical germ cells confined to the seminiferous tubules, with preservation of the basal membrane and absence of stromal invasion. In all cases, the tumor cells were round or slightly polygonal, large, with varying amounts of cytoplasm and round to oval nuclei, and occasionally with one or two prominent nucleoli. Mitoses were counted per 2.37 mm^2^ (corresponding to 10 HPF at 400× fields and an ocular FN 22 mm). Mitotic figures ranged from few to numerous (8–20) (Figure 2B, red arrows). The cells had distinct borders, moderately abundant granular cytoplasm, and large, centrally located vesicular nuclei with coarse chromatin and one to two prominent nucleoli. No fibrosis was observed. Necrosis was seen sporadically–as single or multifocal areas. The presence of hemorrhages was also occasionally noted. Leydig cell tumors (ICTs) were the most common component of mixed testicular tumors. They appeared as nodules measuring 1.5 to 4 cm in diameter, macroscopically well-demarcated, loosely arranged, soft, yellow to light brown in color, and often displayed large hemorrhagic or cystic foci–either empty or fluid-filled (Figure 1D–F, yellow arrows). All ICTs had a similar histological structure–composed of round to polygonal cells with finely vacuolated, abundant cytoplasm, small and round eccentrically placed nuclei, and very few mitotic figures, ranging from one to four per HPF (Figure 2C). In most cases, the neoplastic lesions were not in direct contact with each other; they were separated by smaller or larger areas of relatively normal testicular tissue, and thus could be described as not coalescing. In a few cases, where tumors were located close to one another, they were separated by delicate connective tissue (Figure 2D). No mutual infiltration between different tumor types was observed. Occasionally, one tumor focus was surrounded by another, but without histological evidence of intermingling or intratubular development of one cell population within the other. In particular, we did not observe intratubular seminoma developing within a Leydig cell tumor, as described sporadically in the literature.

The average age of dogs in the study group was 11.2 ± 2.1 years. Among the studied dogs, three (11%) showed cryptorchidism, with no difference in frequency compared to the population of 326 dogs with testicular tumors (12%; Chi-square = 0.02, D.F. = 1, *p* = 0.90). In ten cases from the study group, both testicles were examined. In three of these cases (30%), neoplasms were found in both testicles. The frequency of bilateral neoplasms did not differ from that observed in all dogs with testicular tumors (35%; Chi-square = 0.09, D.F. = 1, *p* = 0.77).

The dogs did not differ in age when grouped according to testicular localisation (H = 3.35, D.F. = 2, *p* = 0.19), histopathological diagnosis (H = 4.93, D.F. = 3, *p* = 0.18), or the presence of two or three simultaneous tumor types (Mann–Whitney U = 25.0, *p* = 0.42).

## 4. Discussion

The conducted analysis indicates that multiple tumors within one or, in some instances, both testicles in dogs are not an extremely rare phenomenon [3,4,5,24]. In human and veterinary pathology, the term collision tumor is sometimes used to describe the coexistence of two distinct neoplasms within the same organ [31]. In our study, we preferred the descriptive term “multiple unilateral testicular tumors” to emphasize the independent histogenesis of each lesion rather than a single mixed neoplastic process.

In our study, their occurrence accounted for 8.28% of all testicular tumors. Various combinations of ICT, SEM, and SCT tumors were the most frequently observed in this study. Despite the limited number of cases, it can be concluded that this type of process occurs predominantly in older dogs over 10 years old. Manuali et al., in a retrospective five-year study (2014–2018) involving 388 cases of testicular tumors in a population of 355 dogs in central Italy (Umbria), reported 10% of cases with multiple tumors in the same testicle [13]. The most commonly observed combination was SEM–ICT (14 cases; 63.6%), followed by SEM–SCT (4 cases; 18.2%). Only two dogs were diagnosed with the ICT–SCT combination (9.1%), and only one case showed a combination of all three tumor types: ICT–SEM–SCT (9.1%). In our material, no mixed tumors (e.g., Sertoli cell–germ cell mixed type) were identified. This may be due to strict inclusion criteria—only cases with clearly separable neoplastic populations were analyzed, whereas lesions with histologically mixed features were excluded. In a study by Grieco et al. involving testicular tumors in 232 dogs, 19 cases (8.18%) of multiple tumors within one testicle were identified [3]. Again, the SEM–ICT combination was most common–8 cases (42%). The remaining were ICT–SCT (5 cases; 26%), SEM–SCT (4 cases; 21%), and SCT–ICT–SEM (2 cases; 11%). This research team also reported a noticeable increase in the frequency of testicular tumors in dogs over the past 40 years. According to the cited authors, testicular tumors affected up to 27% of male dogs. In a study conducted in Brazil by Nascimento et al., among 190 dogs diagnosed with testicular tumors, more than one tumor type was identified in 30 of them (15.8%). Sixteen dogs (8.42%) had different tumors confined to the same testicle, and in four cases, different tumors affected both testes [4]. The most frequent tumor combination was SEM and ICT, observed in 16 dogs, followed by SEM and SCT (12/30), and SCT and ICT (2/30). Liao et al., in a 12-year retrospective study, reported the occurrence of multiple tumors in one or both testicles in 16.8% of cases (80/476) [32]. Multiple unilateral tumors were observed in 22 patients (4.62%). Among them, the SCT–SEM combination was diagnosed in 12 cases, five cases showed SEM and ICT, four presented SCT and ICT, and one tissue sample contained all three tumor types (ICT, SEM, and SCT). The highest percentage of multiple tumors within a single testicle was reported by Slovenian researchers [5]. They described a 17-year study involving 206 dogs and 301 testicular tumor cases, among which as many as 37 cases (18%) featured two or more different tumor types located unilaterally. The team documented co-occurrence of SEM–ICT 12 times (32.4%), SEM–SCT 9 times (24.3%), SCT–ICT 2 times (5.4%), and SEM–SCT–ICT in 2 dogs (5.4%). Bilateral tumors were found in 28 dogs (13.6%), of which, twelve dogs (5.8%) had one tumor in each testicle, but notably–in the context of this analysis—16 dogs (7.8%) had multiple tumors in both testes, appearing in the various combinations mentioned above. Compared with the results cited above, our dataset also demonstrates the predomination of the SEM–ICT combination. Within our study the other combinations were noted with equal frequency—each occurring 6 times (22.22%). The concurrence of all three tumor types (SCT–SEM–ICT) was recorded in three cases (11.11%), slightly more frequently than in the Slovenian study. Our findings corroborate the results described by the other research teams above. According to the cited studies, multiple tumors within the same testicle were found in 4.62% to 18% of examined cases, while in our study, the incidence was 8.28%. The distribution of tumor types in our retrospective analysis also closely aligns with the previously cited reports. In the aforementioned studies, the combination of seminoma and Leydig cell tumor (SEM–ICT) was most prevalent, this was also the most frequent within our study (10 cases/37.04%). Similarly to the previously noted authors, we did not find deviations in the histological architecture of the examined tumors. There was also a lack of observable malignancy to any significant degree, compared to solitary tumors of the same type described in the pathology literature. Cases of unilateral, multiple primary testicular tumors also occur in men. A study by Cicero et al., who compiled data from seven different centers in the USA, found only 1.83% (9/492) of cases of unilateral tumors with differing histology [7]. According to other data, however, these account for between 12% and 22.83% of examined lesions [8,9]. Cicero et al. emphasized that the recognition of lesions with different echostructures may assist pathologists in properly selecting biopsy sites to determine the nature of each lesion [7].

Results obtained in the group of non-multiple testicular tumors show that the most frequently diagnosed testicular tumors were seminomas (38.46%) and interstitial (Leydig) cell tumors (37.12%), while Sertoli cell tumors accounted for 18.39% of cases. The distribution of tumor types broadly corresponds to previously published data, although some differences were observed. Grieco et al. reported a predominance of interstitial cell tumors (approximately 50%) and a lower proportion of Sertoli cell tumors (8%) [3]. Similar results were obtained by Manuali et al., who noted 50%, 29%, and 17% of interstitial, seminoma, and Sertoli cell tumors, respectively [13]. In contrast, Švara et al. found seminomas to be the most common (47.8%), followed by interstitial (28.6%) and Sertoli cell tumors (19.6%) [5]. Nascimento et al. reported a slightly higher frequency of Sertoli cell tumors (27.7%) with a comparable incidence of seminoma (40%) [4].

Differences among studies may reflect variations in sampling methods (necropsy vs. clinical or registry material), breed composition, and age structure of the examined populations. In the present material, cryptorchid testes accounted for 8.58% of all examined gonads, which is somewhat higher than the 5.9% reported by Manuali et al. [13]. As in previous studies, Sertoli cell tumors predominated among neoplasms arising in undescended testes. Bilateral testicular tumors were diagnosed in 7.97% of dogs, a proportion comparable to that observed by Švara et al. 13.6%, considering that both testes were submitted for histopathological evaluation only in a subset of animals [5].

Although no statistically significant associations were observed between age, tumor localization, or the presence of multiple tumor types, these findings are consistent with previous reports indicating that such variables are not major determinants of testicular tumor development in dogs. The similar frequency of cryptorchidism and bilateral tumors between our study group and the broader population further supports the notion that these characteristics do not substantially modify the risk of multiple neoplasms.

The presence of multiple lesions is considered, in human medicine, an absolute contraindication for testis-sparing surgery. It is noteworthy that, even among diffuse seminomas with higher mitotic indices, no vascular or lymphatic emboli were detected. This may be related to the limited invasiveness of these lesions and the early detection of most cases. A limitation of the study is that a single pathologist performed all histological evaluations. Future work should include independent verification to improve diagnostic consistency.

## 5. Conclusions

This study provides data on the frequency and histopathological features of multiple primary testicular tumors within dogs. We confirm that co-occurring tumors show morphological characteristics typical of their respective solitary counterparts and do not exhibit increased atypia and/or malignancy. We acknowledge the need for further observations and research, as well as efforts to explore the role of various risk factors—such as age, diet, environmental pollution, physical activity, and testicular trauma—in the pathogenesis of not only multiple tumors but also testicular neoplasms in dogs in general. Although clinical data were unavailable, our findings may help pathologists and clinicians recognize the frequency and morphology of multiple testicular tumors in dogs.

Future studies should focus on identifying molecular and immunohistochemical markers that could elucidate the biological relationships among coexisting testicular tumors. The use of markers such as Ki-67, vimentin, inhibin-α, or anti-Müllerian hormone may help clarify whether multiple tumor types arise independently or share common oncogenic pathways. Moreover, prospective studies integrating clinicopathological and environmental data could contribute to understanding the role of age, hormonal status, and exposure to environmental pollutants in testicular tumorigenesis in dogs.

## Figures and Tables

**Table 1:** Combinations of testicular tumors occurring together in the described cases (seminoma-SEM, Sertoli cell tumor-SCT, Interstitial cell tumor-ICT, leydigoma).

No.	Breed and Age of the Dog	Testicle Location	Histological Diagnosis	Condition of the Other Testicle
1.	Poodle, 13 years old	In the scrotum	SEM-ICT	Without changes
2.	Boxer, 10 years 8 months	In the abdominal cavity	SEM-ICT	Not tested
3.	Golden retriever, 8 years 6 months	In the scrotum	SCT-ICT	Not tested
4.	Yorkshire terrier, 10 years old	as above	SEM-SCT	SEM-ICT
5.	Jack Russel terier, 12 years, 2 months	as above	SCT-ICT	Without changes
6.	Yorkshire terrier 10 years old	as above	SEM-SCT	Not tested
7.	Beagle, 11 years 1 month	as above	SEM-ICT	ICT
8.	Mixed breed, 13 years 3 months	as above	SEM-ICT	SEM
9.	Beagle 11 years 11 month	as above	SEM-ICT-SCT	Without changes
10.	Welsh corgi pembroke 7 years	as above	SEM-ICT	Not tested
11.	Mixed breed, 15 years	In the inguinal canal	SCT-ICT	Without changes
12.	Mixed breed, 12 years 11 months	In the scrotum	SCT-ICT	Not tested
13.	Maltese, 13 years	as above	SEM-SCT	Not tested
14.	Welsh Terrier, 11 years	as above	SEM-ICT	Not tested
15.	Mixed breed, 11 years	as above	SEM-ICT	Not tested
16.	Mixed breed, 13 years	as above	SCT-SEM	Not tested
17.	Kangal, 12 years	as above	SCT-SEM	SCT
18.	Shih tzu, 13 years 8 months	In the abdominal cavity	SCT-ICT	Not tested
19.	Yorkshire terier, 11 years 1 month	In the scrotum	SEM-ICT	Not tested
20.	German shepard 7 years	In the scrotum	SEM-ICT	Not tested
21.	West highland white terier, 12 years	as above	SEM-ICT	Not tested
22.	Mixed breed 13 years 7 months	as above	SCT-ICT	Not tested
23.	West highland white terier, 9 years 7 months	as above	SCT-ICT-SEM	Without changes
24.	Mixed breed 10 years 9 months	as above	SCT-ICT-SEM	Without changes
25.	Ciarn terier 9 years 2 months	as above	SEM-ICT	Not tested
26.	Mixed breed, 12 years	as above	SEM-ICT	Not tested
27.	Golden retriever 7 years 6 month	as above	SEM-SCT	Not tested

## Data Availability

The raw data supporting the conclusions of this article will be made available by the authors on request.

## Abbreviations

The following abbreviations are used in this manuscript:

SEM	seminoma
SCT	Sertoli cell tumor, sertolioma
ICT	Interstitial cell tumor, Leydigoma
HE	Hematoxylin/eosin
HPF	High Power Field
FN	Field Number
